# Numerical Simulation of Uniaxial Compressive Strength and Failure Characteristics of Non-Uniform Water-Bearing Sandstone

**DOI:** 10.3390/ma16196396

**Published:** 2023-09-25

**Authors:** Mingwei Song, Wenzhi Zhang, Youfeng Zou, Junjie Chen

**Affiliations:** 1 School of Surveying and Land Information Engineering, Henan Polytechnic University, Jiaozuo 454000, China; chsongmw@126.com (M.S.); zouyf@hpu.edu.cn (Y.Z.); chenjj@hpu.edu.cn (J.C.); 2 Henan Engineering Research Center of Ecological Restoration and Construction Technology in Goaf Site, Jiaozuo 454000, China

**Keywords:** non-uniform sandstone, Weibull distribution, uniaxial compression, water-containing state, mechanical properties

## Abstract

As complex and heterogeneous materials, the mechanical properties of rocks are still in need of further investigation regarding the mechanisms of the effects of water. In engineering projects such as goaf foundation treatment and ecological restoration, it is particularly important to describe the fracturing process of non-uniform water-containing sandstone media. The study utilized the theory of continuum mechanics to adopt an elastoplastic strain-softening constitutive relationship and develop a numerical model for analyzing the uniaxial compressive strength and failure characteristics of non-uniform water-containing sandstone. The results indicate that, compared with the reference rock sample, the shorter the capillary path of water entering the rock sample’s internal pores or the larger the contact area with water, the shorter the time required for the rock sample to be saturated. Increasing the water content causes a rapid decline in the rock sample’s elastic modulus and intensifies its brittleness. Group D2 and D3 samples exhibited a decrease in average peak strength to 70.4% and 62.1%, respectively, along with a corresponding decrease in the elastic modulus to 90.78% and 76.55%, indicating significant strain softening. While the failure mode of the rock sample remains consistent across different water contents, the homogeneity of failure shows significant variation. Increasing volumetric water content raises the likelihood of interconnecting cracks between rock samples, resulting in a progressive decline in macroscopic mechanical properties such as peak strength, critical strain, and elastic modulus. This research is significant in advancing the theory and construction technology for ecological restoration in goaf areas.

## 1. Introduction

The occurrence of sudden rock failure during mining production poses a significant threat to the safety of the mine operation. Rocks, being a typical heterogeneous material, display notable variations in properties like density, porosity, elastic modulus, and strength, which are closely associated with their water content. In practical engineering, rocks commonly exist in a water-containing state, and their mechanical properties are significantly influenced by environmental conditions, including a size effect [1].

Presently, numerical simulation methods are widely employed by both domestic and international scholars to investigate damage issues in non-uniform media. Many researchers have focused on studying the mechanical characteristics of non-uniform rock samples by analyzing the geometric parameters of mineral particles. Discrete element and hybrid element methods, such as PFC2/3D [2], UDEC [3], and ELFEN [4], typically model the formation of mineral crystal boundaries and cracks within rocks by defining inter-particle contact. Lan et al. [5] employed UDEC (a 2-D numerical program based on the distinct element method (DEM) for discontinuum modeling) to investigate how the distribution of mineral grain sizes affects stress distribution and crack propagation in the model. Tang et al. [6] introduced spatial correlation scale factors to describe the spatial distribution characteristics of minerals and discovered their significant influence on rock failure morphology and mechanical behavior. Luo et al. [7] proposed a novel parameter assignment method based on the type and content of minerals in non-uniform rock materials. They analyzed the impact of microscale mechanical parameters on the macroscopic mechanical parameters of rocks. Gao et al. [8,9] developed a crystal breakable model using UDEC to simulate the macroscopic and microscopic fracture characteristics of non-uniform brittle sandstone. Pan et al. [10] developed a hybrid cohesive model to investigate the influence of mineral particle size, distribution, and preferred orientation on the mechanical behavior and failure mode of brittle rocks. In cases where the rock material exhibits high homogeneity, shear dilation or brittle failure is more likely to occur. The spatial distribution of rock material particles in contact directly impacts the mechanical response characteristics of the material. Weaker areas tend to experience yielding phenomena, resulting in progressive failure or a softening effect. Although the aforementioned studies have produced valuable insights regarding the non-uniformity of minerals, they have not extensively investigated and described the distribution characteristics of rock heterogeneity, as well as the overall mechanical properties and fracture characteristics of rock samples under the influence of water.

Several scholars have employed micromechanics in studying rock fracture problems. They utilize statistical distribution functions to describe the physical and mechanical properties of rock materials at the microscopic scale. Additionally, they adopt the elastic damage or elastoplastic theory from continuum mechanics to describe the mechanical response of microstructural units. Weibull et al. [11,12] discovered that mechanical parameters of rocks, such as tensile strength, compressive strength, and shear strength, follow a specific statistical distribution law. Gao et al. [13] provided theoretical proof that the shape parameter of the Weibull distribution serves as a measure of the irregularity degree of defect distribution within the material structure. Tang et al. [14,15,16] were the first to introduce the Weibull distribution as a hypothesis into numerical models and investigate the brittle fracture characteristics of two-dimensional rock models. Chen et al. [17,18] noted that simulation methods based on micromechanical continuum mechanics align with the cross-scale fracture characteristics observed in real rocks. Zhang et al. [19] demonstrated the appropriateness of employing the Weibull distribution to construct a micromechanical probability element in elastic damage analysis. Wang et al. [20,21] utilized the Weibull distribution to characterize the non-uniformity of rocks. They investigated the influence of heterogeneity on the mechanical properties and failure characteristics of rocks. Their findings revealed a statistical distribution model capable of describing rock heterogeneity. The research hypotheses put forth by these researchers suggest that material uniformity directly impacts the peak strength and elastic modulus of numerical materials. However, the verification of the Weibull distribution model’s applicability has not been conducted.

Tang et al. [22] introduced spatial correlation scale factors and considered the spatial correlation characteristics of rocks to improve the Weibull distribution model. Zhou et al. [23] utilized the elastic–brittle constitutive relationship to describe the mechanical behavior of microscopic particles and employed the Weibull distribution to characterize the non-uniformity of rocks, simulating the failure of anisotropic elastic–brittle rocks. With increasing heterogeneity, the change in peak strength becomes more pronounced, and the stress–strain curve displays enhanced nonlinear characteristics. Upon crack formation and propagation, shear persists until failure, resulting in a sequential progression of tensile, shear, and plastic flow failures in the rock sample. Generally, the statistical distribution characteristics of certain fundamental physical and mechanical properties of rocks need to be determined based on a large number of laboratory experiments using statistical analysis and hypothesis testing methods. Considering the intricacy of the natural diagenesis process, the utilization of the Weibull distribution to characterize the non-uniformity of rock materials proves to be highly applicable [24,25]. However, the computational models proposed in these studies solely involve elastic deformation, lacking plastic strain throughout the entire process of deformation and failure of microscopic units. Despite allowing for plastic deformation of microscopic units, the EPCA3D model fails to capture their subsequent yielding behavior. Liu et al. [26,27] also employed the Weibull distribution to describe the heterogeneity of rock media, but they utilized an elastic–plastic strain-softening constitutive model to describe the subsequent mechanical response of mesoscopic units. Among these models, the Weibull distribution is better suited for describing the true distribution characteristics of mechanical parameters in rock materials.

The mechanical properties of rocks are also greatly affected by water [28,29], and numerous studies have investigated their behavior under simple loading conditions [30,31]. Rajabzadeh et al. [32] discovered a significant positive correlation between porosity, rock type, and the uniaxial compressive strength of saturated and dry sedimentary limestone and marble. Li et al. [33] proposed a comprehensive dynamic stress–strain constitutive relationship that describes both the pre-peak hardening stage and post-peak softening stage. Zhu et al. [34,35] investigated the impact of various pre-peak unloading damages on the dynamic and static mechanical properties of rocks. Fathi et al. [36] elucidated the influence of cyclic loading on shear mechanisms and shear strength parameters. Zuo et al. [37,38] developed a series of models to investigate the axial crack evolution, crack propagation, and stress–strain relationship of rock masses prior to reaching the peak value. While these studies did analyze the failure characteristics of rock samples during the pre-peak hardening stage and post-peak softening stage, they did not specifically account for the influence of rock heterogeneity. Through laboratory experiments and discrete element simulations, Xiao et al. [39] observed that as the *σ*_1_ level increases, the failure mode of the specimen transitions from mixed tensile-shear failure to shear failure. Additionally, they found a strong correlation between the formation of large cracks in rock samples and the material heterogeneity and true triaxial stress state. Cao et al. [40] employed the Brazilian splitting method to ascertain the mechanical properties of rocks with pre-existing cracks under tension conditions. They discovered that rocks with pre-existing cracks exhibit distinct anisotropic characteristics, with the lowest tensile strength observed when the crack inclination is 45°. These studies observed a correlation between the failure characteristics of rock samples and material heterogeneity under various stress states. However, they did not take into account the influence of water and changes in the effective stress of the material unit. Kewalramani et al. [41] investigated the porosity of concrete containing supplementary cementitious materials by analyzing the volume of permeable voids and conducting water immersion tests. They also examined the water absorption and rapid chloride permeability levels of three different concrete mixtures. Sun et al. [42] developed a numerical model to assess the long-term durability of offshore concrete structures, specifically considering the simultaneous transport of chloride ions and sulfate ions. Golewski investigated the relationship between important physical properties of concrete containing fly ash (CFA) and varying proportions: 0% (CFA-00), 20% (CFA-20%), and 30% (CFA-30%), through water absorption tests. The results indicated that the strength of the material was directly proportional to its water absorption level when CFA was added to the concrete [43]. What are the differences in dynamic failure characteristics and phenomena of rocks under water-containing conditions compared to conventional conditions? The presence of pore water pressure leads to alterations in the effective stress of material units. Currently, these issues still need to be resolved urgently. Revealing the differences in the mechanical responses and failure modes of rocks under different water contents and water locations holds significant importance in preventing and controlling engineering rock disasters in water-containing conditions.

While the Discrete Element Method (DEM) is capable of generating plastic deformation in microscopic structural elements, it fails to capture their subsequent yield behavior. This paper aims to employ the Finite Difference Method (FDM) as the computational framework and utilize the elastic–plastic strain-softening constitutive model based on the Mohr–Coulomb criterion to depict the mechanical response characteristics of microscopic structural elements. This model can describe not only the initial yield process of microscopic structural elements but also their subsequent yielding process. Describing the fracturing process of non-uniform water-containing sandstone media is of particular importance in engineering projects, such as goaf foundation treatment and ecological restoration. This study has important research significance for advancing the theory and improving the construction technology related to ecological restoration in goaf.

## 2. Materials and Methods

### 2.1. Rock Sample Preparation and Testing Methods

**Figure 1:**
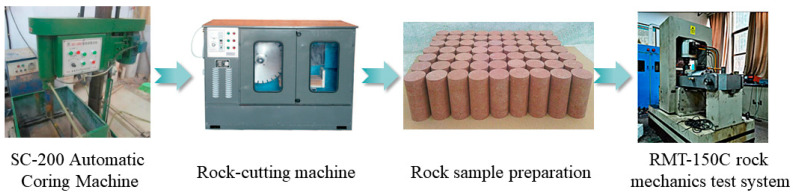
Rock sample production process.

The test was conducted in accordance with the “Standard Test Method for Engineering Rock Masses” (GB/T50266-2013) [44]. The rock sample was obtained from a construction site in Xiuwu County, Jiaozuo. The rock samples had a diameter of 50 mm, and their heights were divided into four groups: 35 mm, 50 mm, 75 mm, and 100 mm. Two rock samples were made for each group and labeled as A1, A2, B1, B2, C1, C2, D1, and D2. Initially, the rock samples were weighed, then dried in a drying oven at 10 °C for 24 h. The four sets of samples were placed in four water tanks, each with a water level height that covered the height of the two rock samples in each group. The rock samples were removed from the water tanks at hourly intervals, and their weights were recorded to determine the water content at different time points. Based on the rock sample from group D with the highest water absorption capacity (D × H = 50 mm × 100 mm), rock samples in three states (dry, natural, and saturated) represented by D1, D2, and D3, respectively, were chosen for uniaxial compression tests. The tests aimed to investigate the effect of different water contents (0%, 0.145%, and 1.6%) on the mechanical properties of sandstone rock samples. To reduce variability, three sets of tests were performed for each state, and the average values were calculated. The RMT-150C rock mechanics test system was utilized for loading, and the deformation rate was set to 0.005 mm/s. The process is shown in Figure 1.

### 2.2. Strain-Softening Constitutive Model

**Figure 2:**
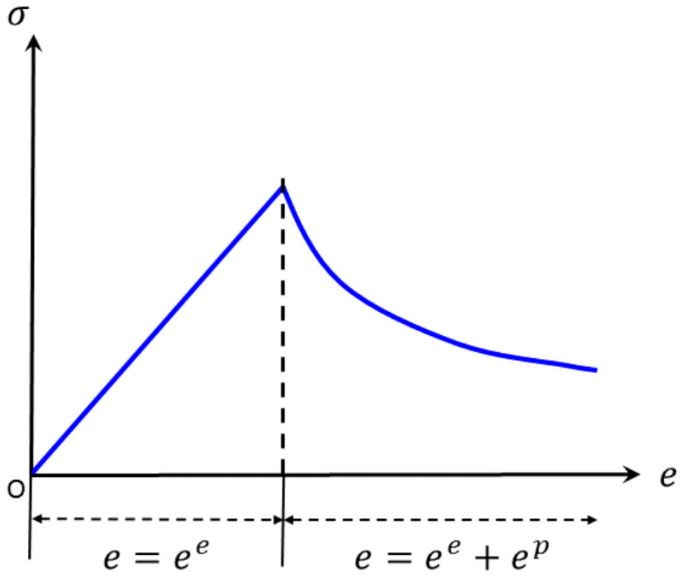
Stress–strain curve of strain-softening material.

The strain-softening model based on the Mohr–Coulomb model has non-associated shear and associated tensile flow rules. It exhibits pre-peak characteristics similar to the Mohr–Coulomb model. However, it demonstrates sudden brittle failure or staged softening and failure after reaching the peak. This behavior is believed to better represent the actual characteristics of rocks, as illustrated in Figure 2.

As the Mohr–Coulomb criterion overestimates the tensile strength of rocks, the yield function is considered with tension truncation [45]. The yield function *f_s_*, *f_t_* is given using

(1) $$\left\{\begin{array}{l} \begin{array}{l} N_{\varphi }=\frac{1+\sin\varphi }{1-\sin\varphi }\\ f_{s}=\sigma_{1}-\sigma_{3}N_{\varphi }+2c\sqrt{N_{\varphi }} \end{array}\\ f_{t}=\sigma_{t}-\sigma_{3} \end{array}\right.$$

where *σ*_1_ and *σ*_3_ are the maximum and minimum principal stresses, *φ* is the internal friction angle, *c* is the cohesive force, and *σ_t_* is the tensile strength.

The mechanical response of the microscopic element is described via elasto-plasticity, which takes into account the post-peak softening characteristics. During the entire process of deformation and failure of the microscopic element, both elastic and plastic deformation occur. The cumulative equivalent plastic shear strain *κ^s^* and cumulative plastic tensile strain *κ^t^* are used as the post-peak strength-softening parameters (internal variables). The incremental form of their definition is given using the equation [45]:

(2) $$\Delta \kappa^{s}=\frac{1}{\sqrt{2}}\sqrt{{\left(\Delta \varepsilon_{1}{}^{ps}-\Delta \varepsilon_{m}{}^{ps}\right)}^{2}+{\left(\Delta \varepsilon_{m}{}^{ps}\right)}^{2}+{\left(\Delta \varepsilon_{3}{}^{ps}-\Delta \varepsilon_{m}{}^{ps}\right)}^{2}}\;\Delta \varepsilon_{m}{}^{ps}=\frac{1}{3}\left(\Delta \varepsilon_{1}{}^{ps}+\Delta \varepsilon_{3}{}^{ps}\right)\;\Delta \kappa^{t}=\left|\Delta \varepsilon_{3}{}^{pt}\right|\;\Delta \varepsilon_{1}{}^{ps}=\lambda^{s},\Delta \varepsilon_{3}{}^{ps}=-\lambda^{s}N_{\psi },\Delta \varepsilon_{3}{}^{pt}=\lambda^{t}$$

In the equation, Δ*ε*_1_*^ps^* and Δ*ε*_3_*^ps^* are the plastic strain increments caused by shear yield; Δ*ε*_3_*^pt^* is the plastic strain increment caused by tensile yield; and *λ^s^* and *λ^t^* are the plastic scalar factors.

The initial yielding surface continuously shrinks as the softening parameter develops, eventually forming subsequent yielding surfaces until the final residual failure surface. The plastic parameter is a non-negative scalar that characterizes the history of plastic deformation in rocks and can serve as an indicator to measure the extent of internal damage development. In the strain-softening model, the plastic parameter describes the dependence of rock strength on plastic deformation. The non-homogeneity of rocks, as discovered by Weibull [11] and Hudson et al. [12], is the main cause of the nonlinear mechanical behavior under external forces. They discovered that the tensile strength, compressive strength, and shear strength of rocks exhibit statistical distribution patterns, validating the heterogeneity of rock media experimentally. The elastic modulus and uniaxial compressive strength values can be determined through uniaxial compression tests on standard laboratory rock samples and employed as non-homogeneous parameters in numerical models. This parameter has been experimentally confirmed to exhibit statistical distribution characteristics by numerous scholars worldwide. Hence, to simplify analysis by reducing non-homogeneous parameters, it is reasonable to set the Poisson’s ratio as constants (as in the studies by Zhou et al. [23] and Feng et al. [25]) and assume that the shear dilation angle, cumulative equivalent plastic shear strain threshold, and cumulative plastic tensile strain threshold of the microscopic unit are proportional to its compressive strength.

The distribution characteristics of the elastic modulus and uniaxial compressive strength of microscopic units are described using Weibull distribution [6,16,17,23,24,25,26,27,28], where the unit cohesion is determined by the uniaxial compressive strength and internal friction angle, while the tensile strength is determined by the compressive strength and the rock tensile/compressive ratio, as shown in the equation [45]:

(3) $$c=\frac{\sigma_{c}(1-\sin\varphi )}{2\cos\varphi }\;\sigma_{t}=\lambda \sigma_{c}$$

In the equation, *σ_c_* is the uniaxial compressive strength, *λ*. The tensile/compression ratio is generally 1/4 to 1/25.

Our method is to consider that the primary factor causing material damage is the reduction in the effective load-bearing area inside the material, which is called geometrical damage, and the damage variable is defined based on the effective load-bearing area of the material structure. The damage variable can be defined from a microscopic perspective using parameters such as the number of defects, defect volume, or fractal dimension. Assuming that the strength probability distribution of micro-elements statistically follows the Weibull distribution, this method offers a lower computational burden and wider applicability. The expression for this method is as follows [11]:

(4) $$p\left(f\right)=\frac{m}{f_{0}}\cdot {\left(\frac{f}{f_{0}}\right)}^{m-1}\cdot e^{-{\left(\frac{f}{f_{0}}\right)}^{m}}$$

**Figure 3:**
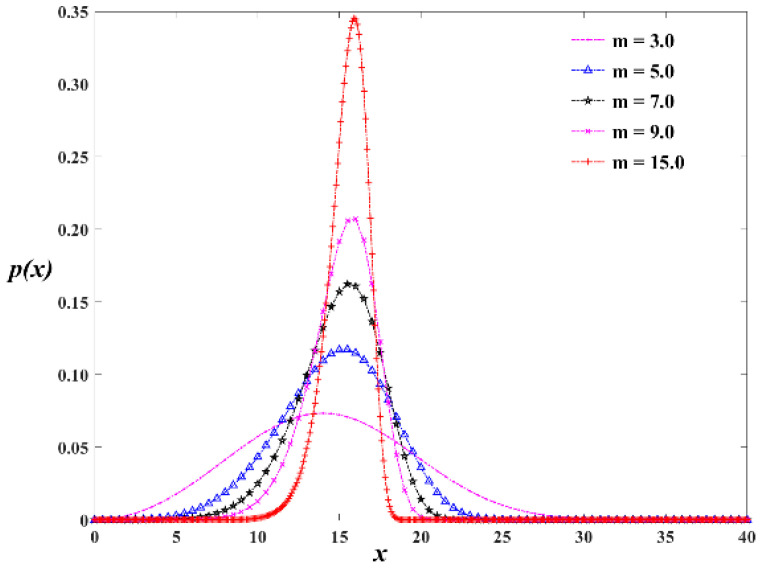
The variation of the Weibull distribution probability density function curve with the *m* value.

In the equation, *p*(*f*) is the probability density, *f* is the mechanical parameter of the rock micro-element, and m and *f*_0_ are the parameters of the Weibull distribution. The probability density function curve of the Weibull distribution for various shape parameters is shown in Figure 3, where m is the shape parameter and *m* > 1 and *f*_0_ is equal to 16. It can be observed that the larger the shape parameter, *m*, the more the material tends to be isotropic internally, while the smaller the *m*, the greater the differences between points inside the material.

The macroscopic failure of rocks is essentially the result of the continuous accumulation of microscopic body damage. The damage variable is defined as the ratio of the number of damaged microscopic elements (*n*) to the total number of microscopic elements (*N*) inside the specimen, and its expression is as follows:

(5) $$D=\frac{\int_{0}^{f}Np(x)dx}{N}$$

According to the Lemaitre strain hypothesis, the damage constitutive equation of the microelement under uniaxial load can be obtained as follows:

(6) $$\sigma =E(1-D)\varepsilon =E\varepsilon e^{-{\left(\frac{f}{f_{0}}\right)}^{m}}$$

When the internal stress of the element satisfies the strength criterion, the strength parameter starts to degrade. In the post-peak stage, the cohesion and shear dilation angle of the microscopic unit gradually soften linearly with the accumulation of the equivalent plastic shear strain, while the tensile strength gradually softens linearly with the accumulation of the plastic tensile strain. The cohesion and shear dilation angle finally soften to the residual value (the product of the initial value and the residual strength coefficient), and the tensile strength eventually decreases to 0. The hardening and softening behavior of the cohesive force, friction force, and dilation force based on the shear parameter Δ*κ^s^* in Formula (2) are presented in a tabular form. This process is implemented through the Fish function. Once the new stress is determined in the calculation step, the hardening parameter of this region is updated according to the above process and embedded in the stress and strain calculation.

### 2.3. Numerical Model Establishment

**Figure 4:**
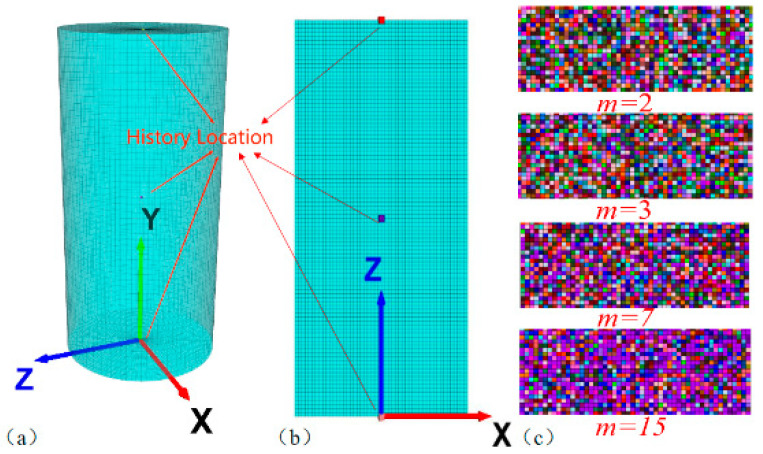
(**a**) Monitoring points of cylindrical numerical model. (**b**) Monitoring points for cylindrical numerical models. (**c**) Distribution characteristics of elastic modulus of materials under different m values.

According to the standards of the International Society for Rock Mechanics for rock uniaxial compression test specimens and research findings from both domestic and foreign scholars, this study employed two numerical models. The first model, referred to as “specimen 1”, was a rectangular prism with dimensions of 100 mm × 50 mm × 1 mm. It consisted of 5000 elements and 5050 nodes, as depicted in Figure 4a. The second model, designated as “specimen 2”, was a cylindrical specimen with a diameter (*D*) of 50 mm and a height (*H*) of 100 mm. It comprised 125,000 elements and 127,551 nodes, as shown in Figure 4b.

The mechanical parameters of the numerical models were determined in line with Table 1. Elastic modulus and uniaxial compressive strength were represented as proportional parameters following the Weibull distribution. The values for elastic modulus, uniaxial tensile and compressive strength, cohesion, and Poisson’s ratio were obtained through laboratory test results. The residual strength coefficient (Rs), plastic shear strain threshold (*κ_sL_*/10^−4^), and plastic tensile strain threshold (*κ_tL_*/10^−4^) were derived from the software operation manual [45].

The values of various mechanical parameters in the strain-softening model will be reduced after the element fails, and these values will differ across different stages of failure. The values of each parameter in each stage are shown in Table 2 [45].

Both models were subjected to a strain-softening constitutive relationship. For model 1, the boundary conditions involved fixing the displacement in the Z direction before and after the rock sample. On the other hand, model 2 had no constraints on either side, and the loading rate at the top and bottom of the sample in the Y direction was set at 2.5 × 10^−6^ m/s, with a strain rate of 2.5 × 10^−5^ m/s.

To define the element strength parameters, the Weibull distribution was utilized. Different values of the parameter ‘*m*’ represented the random generation of heterogeneous materials with varying degrees of homogeneity within the sample. This study selected six non-homogeneity parameters: *m* = 2.0, 3.0, 5.0, 7.0, 9.0, and 15.0. The objective was to investigate the influence of heterogeneity and the spatial distribution of microscopic elements on the macroscopic characteristics of the numerical samples. Figure 4c illustrates the distribution characteristics of the material’s elastic modulus for different values of ‘*m*’.

## 3. Results and Discussion

### 3.1. Analysis of the Factors Influencing the Macroscopic Characteristics of the Rock Sample

#### 3.1.1. Analysis of Size Effect of Sandstone’s Natural Water Absorption Rate

**Figure 5:**
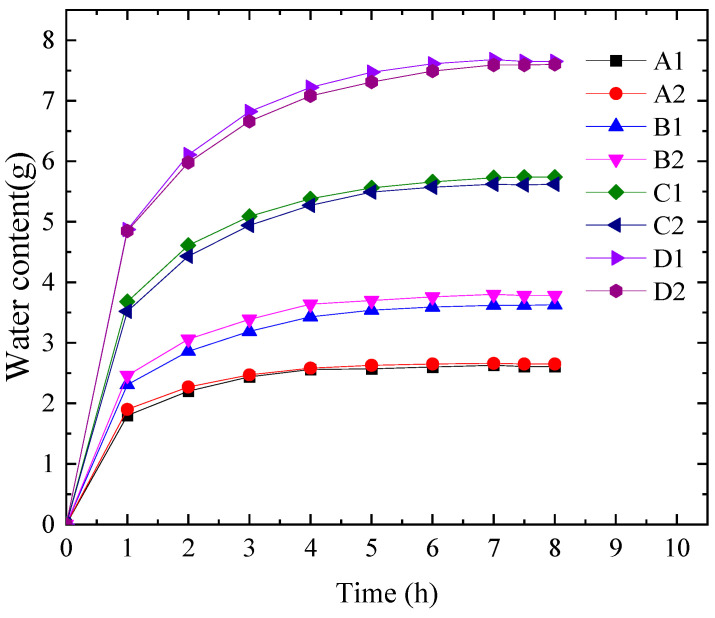
The relationship between water quality content of rock sample and time.

According to the rock sample preparation and testing methods described in Section 2.1, the variation of the water content in the rock samples over time was obtained, as shown in Figure 5.

In Figure 5, a clear correlation can be observed between the water content of the four sets of rock samples at different times. Additionally, different placement methods of the same group of samples have little impact on the water absorption effect of the rock samples. However, it should be noted that the presence of primary cracks within the rock directly affects its mechanical properties. Consequently, the structural porosity and strength characteristics of the rock undergo significant changes when soaked in water [29,30].

**Figure 6:**
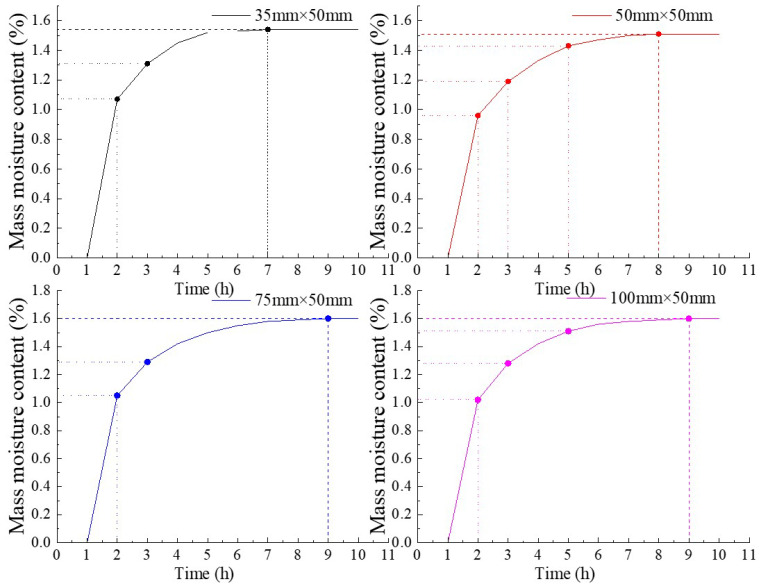
The relationship between the mass moisture content of rock samples with different sizes and time.

The data from eight sets of samples were divided into two groups for comparison. Assuming a constant internal pore capillary suction force, the aspect ratio (H/D) was defined, and the relationship between the water content of different-sized rock samples and time during natural water absorption was analyzed. The mass water content of the rock samples at different times was calculated, and the relationship between the mass water absorption rate of different-sized rock samples and time was plotted, as shown in Figure 6.

From Figure 6, it is evident that the water absorption rates of the three groups of rock samples, relative to the reference group B, are higher in the first two stages. This indicates that the mass water content change in sandstone exhibits a size effect. Specifically, during the initial stage, the water absorption rates of all four sizes of rock samples show an approximately linear relationship. This suggests that in the rapid water absorption phase, water molecules come into immediate contact with the surface pores of the rock samples and penetrate the interior of the samples due to water tension. After one hour, the time taken for each rock sample to reach saturation was analyzed. The duration to reach saturation depends on the length of the capillary pathway for water to enter the internal pores of the rock sample and the contact area between the rock sample and water. Consequently, rock samples with shorter capillary pathways or larger contact areas absorb water more quickly and reach saturation in a shorter time. As a result, samples from group A (35 mm × 50 mm) reached saturation first. However, when the height of the rock sample exceeded 50 mm, the time required to reach saturation became approximately the same for all rock samples.

Under the natural water absorption state, the water absorption process of the rock sample is influenced by capillary suction. The capillary phenomenon occurs because when the rock sample is immersed in a liquid, the liquid surface tension generates additional pressure on the contact surface. The calculation formula for the Laplace capillary pressure of a spherical liquid surface is as follows [46]:

(7) $$P_{c}=\frac{\alpha \cdot 2\pi R\cos\theta }{\pi R^{2}}\;\alpha =75.796-0.145t-0.00024t^{2}$$

In the formula, *P_c_* is the additional pressure, *R* is the radius of the rock pore, *α* is the surface tension coefficient of the liquid, *θ* is the contact angle, *t* is the Celsius temperature (°C), and the unit of surface tension is mN/m.

There are small pore channels in the rock sample that form capillaries when immersed in water. The maximum distance of water infiltration along the pores, under the influence of additional pressure, can be calculated according to Jurin’s law using the following formula [46]:

(8) $$h_{c}=\frac{P_{c}}{\rho g}$$

In the formula, *ρ* is the density of the liquid, *g* is the acceleration due to gravity, and *h_c_* is the pressure head under capillary pressure.

Based on the statistical results of this experiment: Firstly, there is no significant difference in water content among rocks of different heights at the same time, during the initial three hours of water immersion, the rock sample with a height of 35 mm exhibits a slightly higher water content, suggesting that water enters any pore inside the rock from multiple surfaces, resulting in a shorter path for water immersion into the sandstone pore, leading to a faster initial water absorption rate and higher initial water content of the rock sample. Secondly, as the size of the sandstone sample increases the time required to reach saturation increases gradually and once the height exceeds 50 mm. A shorter path of water immersion into the internal pores of the rock results in a shorter required saturation time. Thirdly, the ratio of the final saturated water content for the four heights of sandstone is approximately 35:50:75:100, which corresponds to 2.64:3.71:5.68:7.63. In this condition, heterogeneity has minimal impact on the macroscopic characteristics of the rock sample’s natural water absorption rate, but the size effect during the natural water absorption process is pronounced.

#### 3.1.2. Effect of Inhomogeneity on Macroscopic Properties

**Figure 7:**
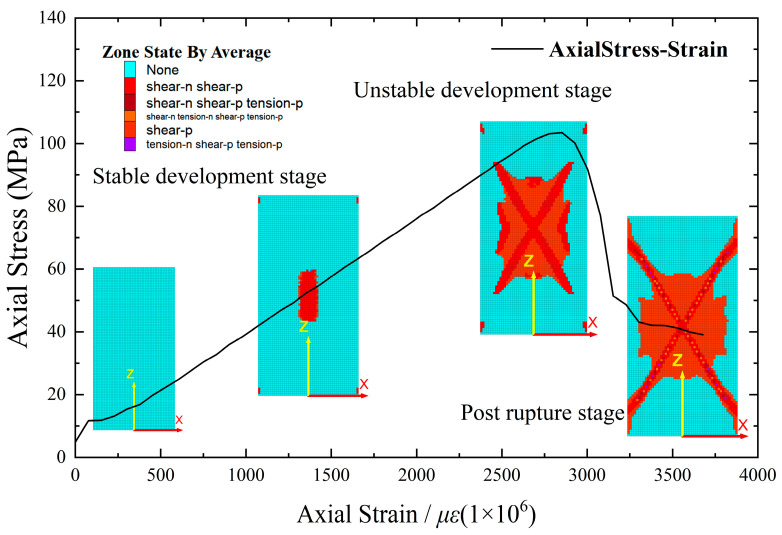
Axial stress–strain curve of the sample and plastic zone at each stage of Model 1.

Model 1 employed cube elements with dimensions of 1 mm × 1 mm × 1 mm. A model with the same material properties for all elements was set as the standard group. The plastic zone range was monitored at intervals of 10 time steps, segments exhibiting a substantial increase in the plastic zone range were identified as turning points, signifying the turning points in the rock’s deformation process at various stages. Consequently, the monitored full stress–strain curve was divided into three distinct stages. Figure 7 illustrates the distribution of plastic zones at different stages.

In the simulation of homogeneous rock samples, the stress–strain curve exhibits a general trend, as depicted in Figure 7, and can be roughly classified into three stages. (1) Stage I corresponds to linear elastic development, characterized by a relatively stable process and an approximately linear curve. The sample is in the elastic stage, and shear cracks start to appear. (2) Stage II represents unstable yielding, marked by a rapid increase in the number of shear cracks during continuous compression. As the axial stress approaches approximately 85% of the peak stress, the upward trend of the curve gradually decelerates. At the peak point, tensile cracks start to develop. (3) Stage III occurs after the peak, characterized by a decrease in axial stress while the strain continues to increase. The sample gradually loses its bearing capacity until reaching residual strength. As the numerical model does not account for the inherent internal defects of the rock, the simulation curve does not exhibit the densification stage. Nevertheless, rock materials are complex composites, exhibiting substantial variations in mineral particle composition and inter-particle bonding. Even for rock samples with identical origin, lithology, or size, the stress–strain curve, peak strength, and rupture characteristics demonstrate noticeable disparities [5,7,10]. Hence, it is imperative to quantify the heterogeneity of rock samples and investigate its impact on both macroscopic and microscopic fracture characteristics.

**Figure 8:**
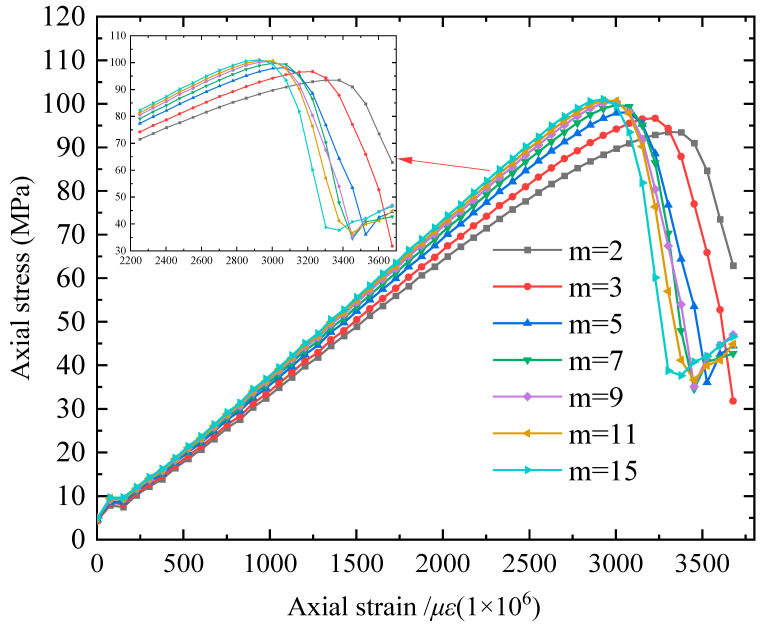
Stress–strain curves of numerical models with different *m* values.

The stress–strain curve of the numerical model exhibits variations with changes in homogeneity parameters under different conditions, as depicted in Figure 8. A larger value of m indicates a higher level of material homogeneity, where the properties within the material tend to be more uniform. Consequently, under the same random variable assignment, the standard deviation of the elastic modulus between elements gradually decreases. This phenomenon suggests that as homogeneity increases, the nonlinear characteristics of the numerical model for the rock sample weaken gradually, while brittleness progressively intensifies. Moreover, when the internal stress of the elements is equal, they are more likely to reach the failure state simultaneously. Conversely, lower homogeneity results in greater dispersion of mechanical parameters among elements, often leading to the strength of the rock being determined by its weakest part and consequently yielding a relatively lower overall strength. These findings support the conclusion that micro-heterogeneity stemming from the non-uniform distribution of grains under uniaxial compression load significantly influences micro-mechanical behavior and macroscopic response. This conclusion has been verified in [10].

**Figure 9:**
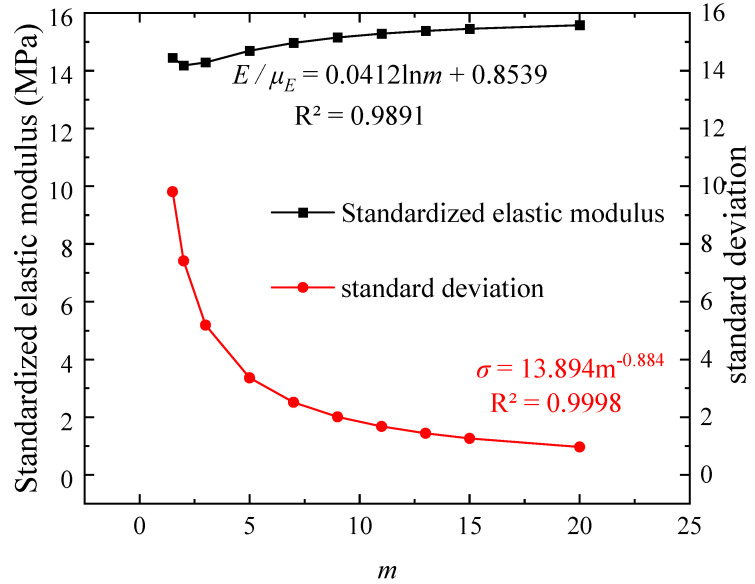
Characteristics of the variation in elastic modulus of material elements in numerical models with different *m* values.

The variation of the elastic modulus of the material elements in the numerical model under different homogeneity parameters is shown in Figure 9. The trend of the numerical specimen’s elastic modulus is evident, transitioning from low homogeneity to high homogeneity (*uE* = 16 MPa). When *m* < 3.0, *E/uE* decreases from 0.90 (*m* = 1.5) to 0.88 (*m* = 2). For 3.0 ≤ *m* < 7.0, *E/uE* increases from 0.89 (*m* = 3) to 0.92 (*m* = 5) and 0.94 (*m* = 7). Once *m* ≥ 7.0, the change in *E/uE* becomes insignificant, stabilizing around 0.96. These phenomena are closely related to the inherent characteristics of the Weibull distribution. Figure 8 presents the fitting curve (*R*^2^ = 0.9891) of the standardized elastic modulus (*E/uE*) and the homogeneity parameter *m*. Tang et al. [6], under the RFPA2D framework, discovered that the relationship between standardized uniaxial compressive strength, standardized elastic modulus, and homogeneity parameter *m* can be expressed as *y* = *a* ln*m* + *b* (9). This formula aligns with the observed behavior of the elastic modulus in Figure 9.

**Figure 10:**
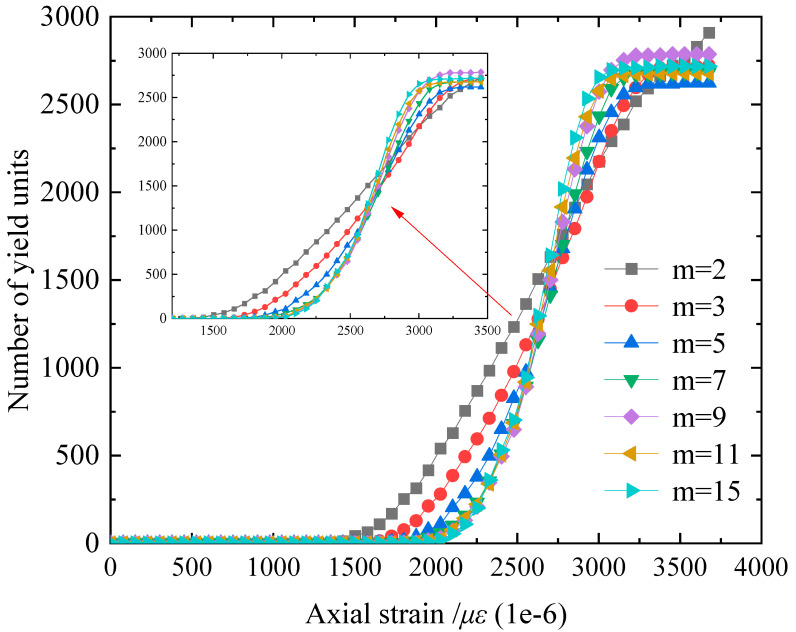
Characteristics of the variation in the initial yield element count with axial strain under different *m* values.

Figure 10 shows the variation characteristics of the initial yield element quantity with axial strain at the material time step. When *m* < 5, the pre-peak stage of uniaxial compression will also produce element yield conditions. However, with the increase in material homogeneity, the time for the appearance of yield elements is delayed. At the strength peak and post-peak stages, the material elements will undergo final yielding, and the number distribution range becomes smaller and tends to be unified. This reflects that as the homogeneity increases, the plasticity of the model gradually weakens, and the brittleness gradually increases. When the micro-homogeneity parameter *m* < 7.0, there is no stress concentration near the peak strength, and the stress–strain curve shows a sharp shape, reflecting that the specimen suddenly loses its bearing capacity after reaching the peak strength and that the strength decreases rapidly, showing typical brittle fracture behavior; for the numerical specimens with *m* ≥ 7.0, whether it is in the pre-peak stage or the post-peak stage, the stress–strain curve of the numerical specimen shows approximate linear characteristics. The stress–strain curve in the pre-peak stage shows an obvious yield feature, and the tangent modulus gradually decreases until it reaches 0 at the peak strength. In the post-peak stage, the strength decreases slowly and exhibits a certain degree of ductility.

**Figure 11:**
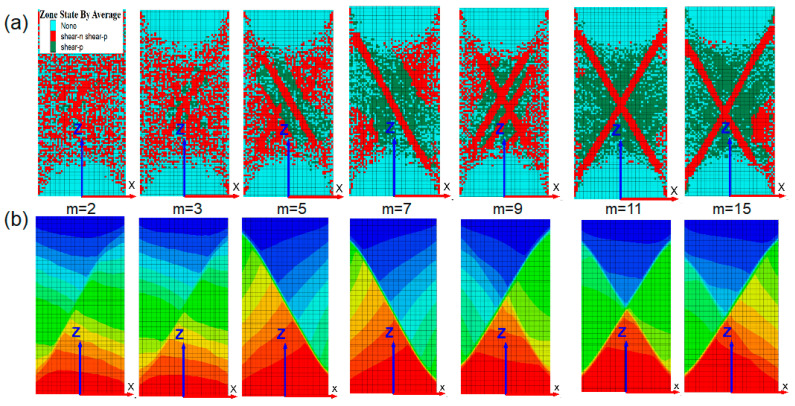
(**a**) The failure modes of numerical rock samples under different m values. (**b**) The z-direction displacement of numerical rock samples under different *m* values.

As shown in Figure 11a, when *m* < 5.0, there is no obvious macroscopic shear zone in the rock sample, exhibiting plastic flow characteristics. Combining its stress–strain curve, a transition failure mode of monoclinic shear occurs after the peak stage. When *m* = 5.0, 7.0, there is a distinct monoclinic macroscopic yielding unit shear zone in the rock sample, with a failure mode of shear along the monoclinic plane. When *m* = 9.0, multiple shear zones intersect and distribute within the rock sample with spacing, and the rock sample begins to exhibit a failure mode transitioning from a monoclinic to X-shaped shear section. When *m* > 9.0, the rock sample undergoes X-shaped double shear failure, with the failure progression developing from the outside inward. Figure 11b shows the z-direction displacement of numerical rock samples after failure under different homogeneities, that is, as the homogeneity increases, the properties inside the material tend to be the same, the nonlinear features of the numerical rock model gradually weaken, and the failure mode changes from monoclinic shear failure to X-shaped double shear failure. Basu et al. [47] conducted a classification of indoor uniaxial compressive failure tests on 76 standard rock samples and identified six significant failure modes. The failure modes occurring along the bedding planes are excluded from the scope of this discussion, taking into account the failure pattern illustrated in Figure 11b.

#### 3.1.3. Probability Distribution Characteristics of Microstructure

**Figure 12:**
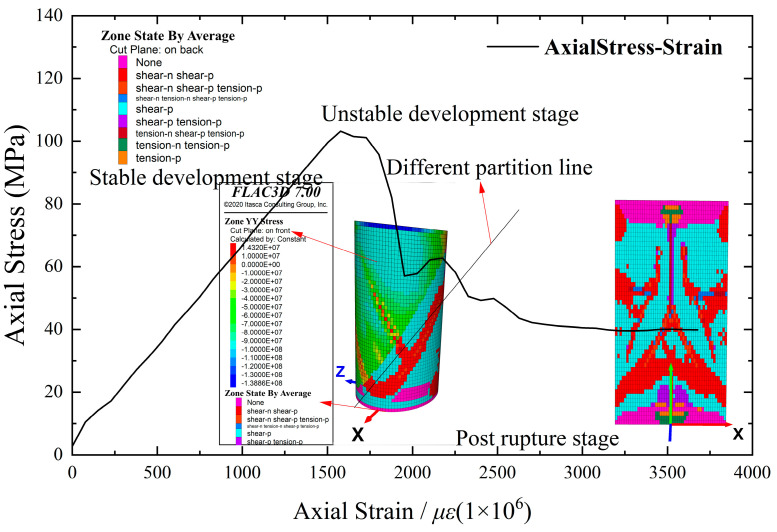
Axial stress–strain curve of the sample and plastic zone at each stage of Model 2.

The plane strain model utilized in this study allows for the analysis of the axial section of the numerical rock sample. As a reference, Model 2 was employed, which maintains consistent material properties across all elements and possesses element dimensions of length × width × height = 1 mm × (0.03–1.57) mm × 2 mm. The boundary conditions are described in Section 2.3, and the distribution of plastic zones at various stages is monitored for every 10 time steps. The distribution of plastic zones at different stages is shown in Figure 12.

**Figure 13:**
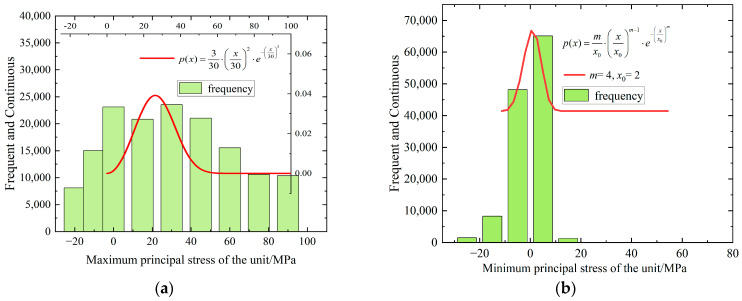
All units during the failure stage. (**a**) Fitting of maximum principal stress distribution. (**b**) Fitting of minimum principal stress distribution.

The stress and strain data of all elements are exported from the post-failure model after segmentation, specifically from the last calculated time step. The SPSS software (version 27) is used for distribution fitting. Through the chi-square test, the best fitting distribution form for the stress or strain data is determined. Figure 13 illustrates the distribution fitting of the maximum and minimum principal stresses in all elements at the final time step of the numerical calculation. On the *X*-axis, the actual stress conditions of all elements are represented, while the *Y*-axis represents the probability density of internal stress in different elements. The curve represents the fitted theoretical probability distribution function. Figure 13a primarily depicts the overall compression situation of the specimen after failure. The upper limit of the interval is positive. Around 84.39% of the elements inside the specimen bear compressive stress, and approximately 59.75% of the elements exhibit a maximum principal stress distribution ranging from 0 to 5 MPa. About 15.61% of the elements experience tensile stress, with 5.46% of the elements surpassing the ultimate tensile strength. This indicates that the main failure mode of the numerical rock sample is compression-shear failure. Despite a significant reduction in compressive capacity, some elements can still withstand a certain degree of compressive stress. Figure 13b mainly portrays the overall tensile situation of the specimen after failure. The 90% confidence interval of the minimum principal stress ranges from −5 to 5 MPa, which does not exceed the ultimate tensile strength. This suggests that the specimen did not completely lose its bearing capacity after failure. Overall, not all elements fail during the failure stage of the specimen. The maximum and minimum principal stresses of all elements in the specimen are separately fitted with probability distributions. After conducting the chi-square test, it is determined that the optimal distribution fitting results for both the maximum and minimum principal stresses follow the Weibull distribution. This confirmation supports the assumption of the Weibull distribution’s rationality for microscopic elements in the plane numerical model.

### 3.2. The Influence Mechanism of Water Physical Properties of Rock Samples

**Figure 14:**
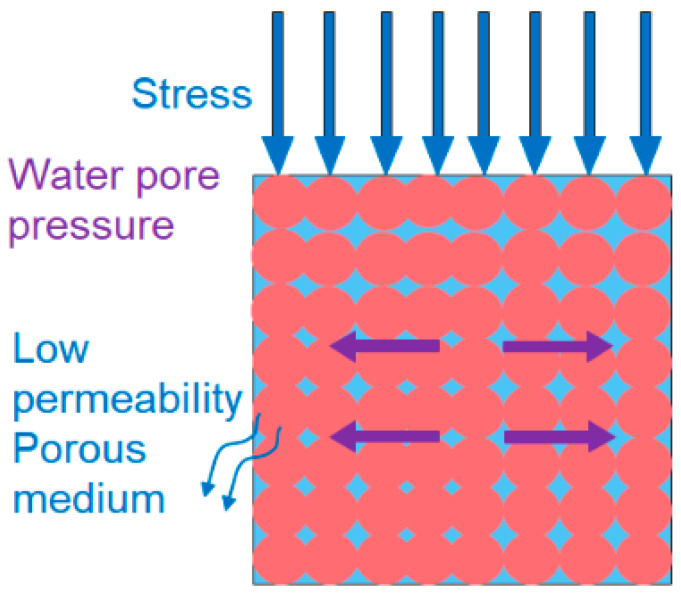
Schematic diagram of effective stress action of rock samples.

The mechanical properties of red sandstone are closely related to the external environment, as well as its water content and degree of weathering. Two main reasons contribute to this phenomenon. Firstly, in the mineral composition of red sandstone, minerals like montmorillonite and kaolinite undergo physical and chemical reactions when exposed to water. These reactions generate an expansion force that causes particles to detach from the binding material. Secondly, the presence of initial cracks in the rock sample makes it vulnerable to the effects of external water environmental conditions. Binding materials such as mud, iron, and calcium within the particles are easily dissolved, resulting in a loss of their binding ability [48]. Due to these two factors, new cracks emerge in the rock sample, which become filled with water and serve as a medium for pressure transmission. Over time, under the influence of the expansion force, these cracks gradually widen and eventually stabilize. Figure 14 provides a visual representation of this process.

**Figure 15:**
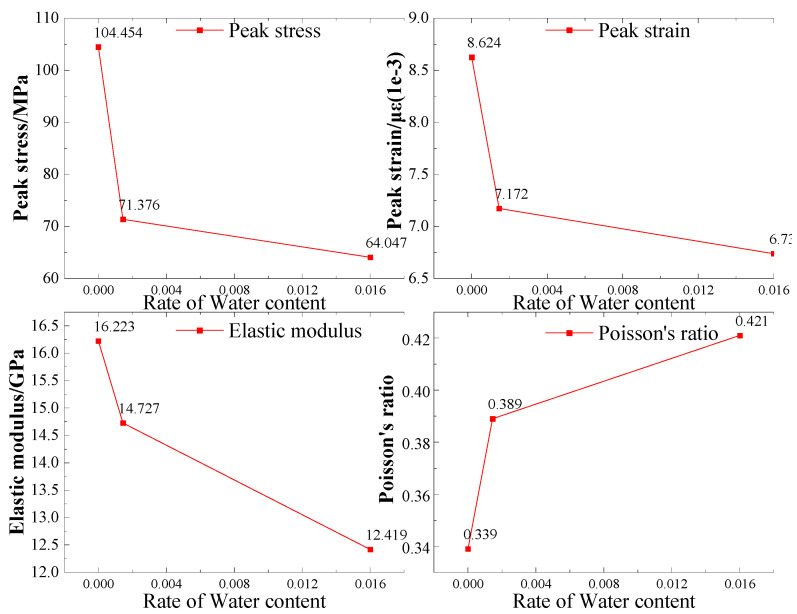
The relationship between mechanical parameters and water content of rock samples under uniaxial compression.

The data obtained from uniaxial compression tests of rock samples under three conditions (dry, natural, and saturated) were collected into a table, and three sets of tests were selected for each condition for comparison. Organize the data in the table and plot them into the figure as shown in Figure 15.

From Figure 15, it can be observed that with the increase in the water content in the rock samples, the uniaxial compressive strength of sandstone decreases rapidly under the same loading rate. Comparing the samples in group D1 with those in groups D2 and D3, the average peak strength of the latter decreased to 70.4% and 62.1%, respectively. Additionally, the elastic modulus decreased to 90.78% and 76.55%, while the peak strain decreased to 83.2% and 78.1%, respectively. One possible reason for these findings is that the initial micro-cracks present within the rock samples continue to develop in a water-containing environment. The filling of water in the pores leads to partial dissolution of the mineral components. As a result, the cementitious material between particles loses its binding ability due to water saturation, resulting in a decrease in the frictional force between the particles. The elastic modulus of the rock samples decreases as the water content increases until macroscopic failure occurs. Another contributing factor may be the direct shear of mineral particles under stress, leading to a significant difference in failure stress between dry and water-saturated samples. However, this type of failure often accompanies violent fragmentation of the rock. In the case of water-saturated red sandstone samples after compression, the softening and decomposition effect of water makes cracks more likely to penetrate each other, increasing the internal pore spacing. This affects the mechanical properties of the sandstone structure, resulting in a strength-softening effect. Consequently, there is no shearing of mineral particles, and the strength of the water-saturated samples is much lower than that of the dry samples. These findings align well with the research results of Zhou [49].

### 3.3. Failure Law of Sandstone under Uniaxial Compression under Different Water Content

Utilizing a plane strain numerical model, under the same conditions of microscopic element properties, equivalent parameters for pore water pressure are assigned according to the position of the water level of the rock sample.

**Figure 16:**
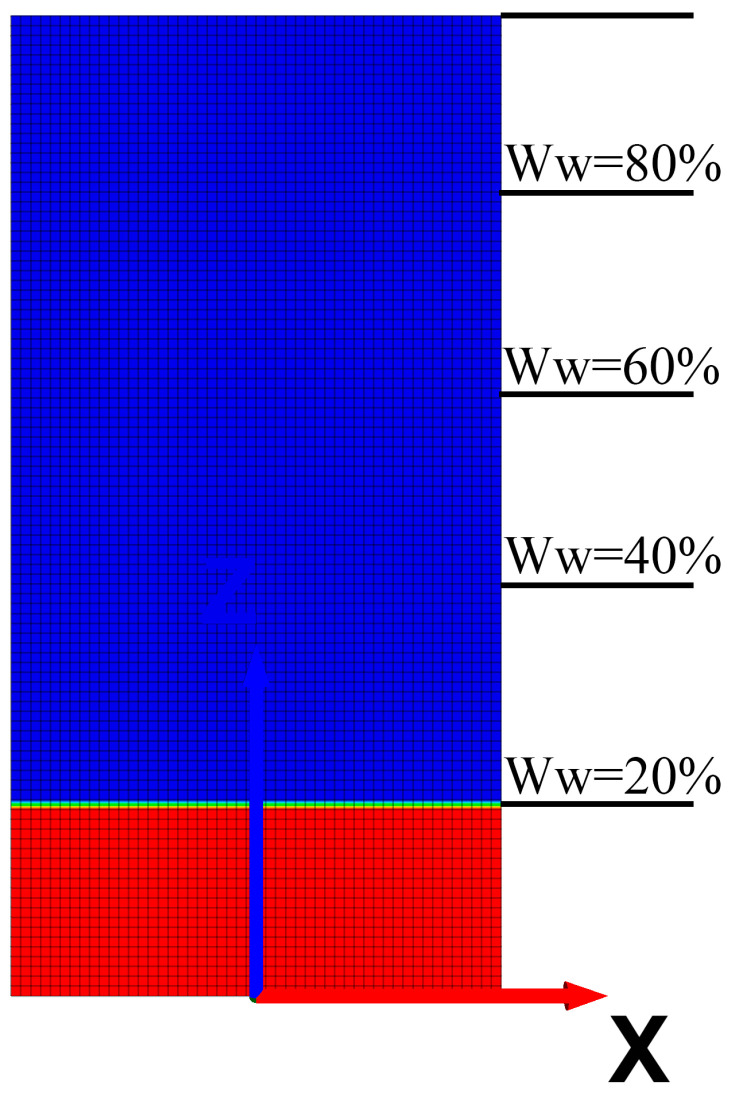
Position relationship of pore water pressure in numerical rock sample units.

Five control groups were established (for volumetric water contents of 20%, 40%, 60%, 80%, and 100%), and the equivalent pore water pressure within the element was assigned according to a gradient below the water level and kept constant, as shown in Figure 16.

**Figure 17:**
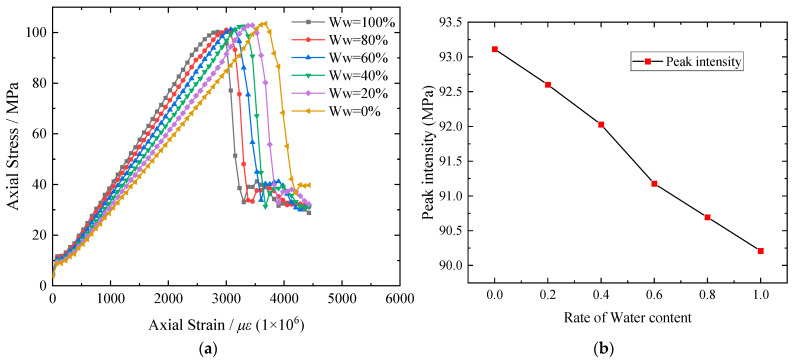
(**a**) Stress–strain relationship curves of rock samples under uniaxial compression under different volume moisture contents. (**b**) Curve of peak strength variation of rock samples under different volume moisture contents.

The stress–strain relationship of rock samples under varying volumetric water contents was determined through numerical calculations of the model, as shown in Figure 17.

From Figure 17a, it is evident that the duration of rock compaction during the saturation process is shorter compared to the drying process. As depicted in Figure 7, the rock undergoes three stages: initial stage, elastic stage, and plasticity stage. During the saturation process, the internal cracks of the red sandstone close in the initial stage, leading to a gradual decrease in elasticity and an increase in plasticity compared to the drying process under the same water content. The rock experiences minimal expansion of internal cracks and generates a few new cracks. The damage variable undergoes slight changes, and the damage stress gradually decreases, indicating a reduced stress requirement for crack generation and expansion. Ultimately, the macroscopic failure of the rock is primarily driven by the continuous decline in peak stress, elastic modulus, and critical strain. Thus, varying water content and load induce different levels of damage to the rock sample. The numerical simulation results align with the laboratory test results before the rock sample reaches critical strain. However, notable discrepancies emerge in the post-peak stage. This discrepancy may arise from the fact that, prior to reaching the critical strain, the stress within the rock sample primarily originates from external loads. When the rock sample is dry, failure occurs through a single oblique crack in the middle. As the water content increases, the softening and decomposing effects of water cause cracks to intersect and intensify fragmentation upon sample damage. Consequently, the failure mode transitions from a single oblique crack to a double oblique section shear failure mode, consistent with the findings of Zhuang [50]. From Figure 17b, it is evident that the peak strength of the rock sample gradually decreases as the water content increases. However, the fluctuation range is minimal, and the relationship between peak strength and water content appears approximately linear. This observation can be attributed to the relatively small size of the rock sample (50 mm × 100 mm) and the limited variations in water content among the different samples. The size effect is pronounced, but its overall impact on the rock sample remains constrained.

## 4. Conclusions

The findings of this study can be concluded as follows:

(1) The water absorption process in sandstone demonstrates a size effect, although the saturation time remains relatively constant when the height of the sandstone sample exceeds 50 mm. As micro-homogeneity increases, the nonlinear behavior of the numerical model gradually diminishes, while brittleness intensifies. The elastic modulus has a linear relationship with ln(*m*), and the heterogeneity of the rock has a significant effect on its internal stress distribution;
(2) As the water content increases, the compressive strength, elastic modulus, and failure strain of the rock sample experience a rapid decrease. The static mechanical properties of the rock sample demonstrate a gradual decline in peak strength, critical strain, and elastic modulus. Additionally, the failure mode gradually transitions from a single oblique surface to a double oblique shear failure form, accompanied by an increased degree of sample fragmentation;
(3) The time required for rock compaction during saturation is shorter than that during drying. The elasticity of red sandstone decreases, and the plasticity increases during saturation. With the increase in volumetric water content, the cracks between rock samples are more likely to penetrate each other, the internal pore spacing increases, and the mechanical properties of sandstone structure are affected, resulting in a strength-softening effect, which is particularly important in decision making for goaf foundation treatment measures.

## Figures and Tables

**Table 1:** Numerical model mechanical parameter values.

Mechanical Parameters	Value
Elastic modulus *E_0_*/GPa	16
*f_s_*/MPa	103
*f_t_*/MPa	15
Poisson’s ratio *ν*	0.34
Internal friction angle *φ*	45
Dilation angle *ψ*	10
Residual strength coefficient *Rs*	0.05
Plastic shear strain threshold *κ_sL_*/10^−4^	5
Plastic tensile strain threshold *κ_tL_*/10^−4^	2

**Table 2:** Values of mechanical parameters at different failure stages.

Mechanical Parameters	Elastic Stage	Unstable Stage	Post-Rupture Stage
Cohesion/MPa	21.3	5	5
Friction/(°)	45	42	40
Dilation/(°)	10	3	0

## Data Availability

Not applicable.

## References

[1] You M., Zou Y. (2000). Discussion on Rock Heterogeneity and Strength Size Effect. Chin. J. Rock Mech. Eng..

[2] Potyondy D.O., Cundall P.A. (2004). A bonded-particle model for rock. Int. J. Rock Mech. Min. Sci..

[3] Gao F., Stead D. (2014). The application of a modified Voronoi logic to brittle fracture modelling at the laboratory and field scale—ScienceDirect. Int. J. Rock Mech. Min. Sci..

[4] Feng F., Li X., Li D. (2017). Modeling of failure characteristics of rectangular hard rock influenced by sample height-to-width ratios: A finite/discrete element approach. Comptes Rendus Mécanique.

[5] Lan H., Martin C., Hu B. (2010). Effect of heterogeneity of brittle rock on micromechanical extensile behavior during compression loading. J. Geophys. Res. Solid Earth.

[6] Tang X., Zhou Y., Zhang C. (2012). Micromechanical model considering spatial correlation scale characteristics and its application. Rock. Soil. Mech..

[7] Luo R., Zeng Y., Du X. (2012). Study on the relationship between macro and micro mechanical parameters of heterogeneous rock materials. Chin. J. Geol. Eng..

[8] Gao F., Stead D., Elmo D. (2016). Numerical simulation of microstructure of brittle rock using a grain-breakable distinct element grain-based model. Comp. Geol..

[9] Wong L.N., Peng J., Teh C.I. (2018). Numerical investigation of mineralogical composition effect on strength and micro-cracking behavior of crystalline rocks. J. Nat. Gas Sci. Eng..

[10] Pan C., Li X., He L., Li J. (2021). Study on the effect of micro-geometric heterogeneity on mechanical properties of brittle rock using a grain based discrete element method coupling with the cohesive zone model. Int. J. Rock Mech. Min. Sci..

[11] Weibull W. (1951). A statistical distribution function of wide applicability. J. Appl. Mech..

[12] Fang Z., Harrison J. (2002). Development of a local degradation approach to the modelling of brittle fracture in heterogeneous rocks. Int. J. Rock Mech. Min. Sci..

[13] Gao F., Xie H. (1996). Statistically fractal strength theory for brittle materials. Acta Mech. Solid. Sini..

[14] Tang C., Kaiser P.K. (1998). Numerical simulation of cumulative damage and seismic energy release during brittle rock failure-part I: Fundamentals. Int. J. Rock Mech. Min. Sci..

[15] Kaiser P.K., Tang C. (1998). Numerical Simulation of Damage Accumulation and Seismic Energy Release During Brittle Rock Failure-Part II: Rib Pillar Collapse. Int. J. Rock Mech. Min. Sci..

[16] Tang C., Liu H., Lee P.K., Tsui Y., Tham L.G. (2000). Numerical studies of the influence of microstructure on rock failure in uniaxial compression. part I: Effect of heterogeneity. Int. J. Rock Mech. Min. Sci..

[17] Chen Z., Tan G., Yang W. (2002). Renormalization study and numerical simulation on brittle failure of rocks. Chin. J. Geol. Eng..

[18] Yang S., Xu W., Wei L. (2004). Statistical constitutive model and experimental study of rock damage under uniaxial compression. J. Hohai Univ. Nat. Sci..

[19] Zhang M., Li Z., Su X. (2005). Probabilistic volume element Modeling for elastic damage analysis of quasi-brittle materials. Chin. J. Rock Mech. Eng..

[20] Wang S., Zhu H., Feng X. (2006). Effect of meso heterogeneity on macro failure form of brittle rock materials. Rock Soil Mech..

[21] Liang Z., Tang C., Zhang Y. (2008). Random probability model of physical and mechanical parameters and failure mechanical behavior characteristics of quasi brittle materials. Chin. J. Rock Mech. Eng..

[22] Tang X., Zhou Y. (2014). Study of rock uniaxial compression deformation and failure character based on an improved Weibull distribution model. Chin. J. Rock Mech. Eng..

[23] Zhou X., Zhao Y., Qian Q. (2015). Numerical simulation of rock failure process in uniaxial compression using smoothed particle hydrodynamics. Chin. J. Rock Mech. Eng..

[24] Yang Z., Ning Y., Lu M. (2016). Characterization of rock heterogeneity modeled by discontinuous deformation analysis method. J. Chongqing Univ..

[25] Jiang Q., Cui J., Feng X. (2017). Randomness statistics and probability distribution estimation of basalt mechanical parameters. Rock Soil Mech..

[26] Liu J., Zhao G., Liang W. (2018). Numerical simulation of uniaxial compressive strength and deformation and fracture law of heterogeneous rock media. Rock Soil Mech..

[27] Liu J., Zhao G., Peng F. (2020). Statistical probability model for mesoscopic mechanical parameters of rock material under elastoplastic strain-softening framework. J. China Coal Soc..

[28] Vásárhel Y.I.B. (2005). Statistical analysis of the influence of water content on the strength of the Miocene limestone. Rock Mech. Rock Eng..

[29] Li D., Wong L., Liu G. (2012). Influence of water content and anisotropy on the strength and deformability of low porosity meta-sedimentary rocks under triaxial compression. Eng. Geol..

[30] Zuo Y., Li X., Zhou Z. (2005). Damage and failure rule of rock undergoing uniaxial compressive load and dynamic load. J. Cent. South Univ. Technol..

[31] Greco O.D., Ferrero A.M., Oggeri C. (1993). Experimental and analytical interpretation of the behaviour of laboratory tests on composite specimens. Int. J. Rock Mech. Min. Sci..

[32] Rajabzadeh M., Moosavinasab Z., Rakhshandehroo G. (2012). Effects of rock classes and porosity on the relation between uniaxial compressive strength and some rock properties for carbonate rocks. Rock Mech. Rock Eng..

[33] Li X., Qi C. (2018). A micro-macro dynamic compressive-shear fracture model under static confining pressure in brittle rocks. Int. J. Imp. Eng..

[34] Zhu Z., Wei L., Meng Q., Jing H., Su H., Fu A. (2019). Experimental Study on Dynamic and Static Mechanical Properties of Pre-peak Unloading Damaged Marble. Chin. J. Rock Mech. Eng..

[35] Zhu Z., Yu L., Li J. (2020). Research on acoustic emission characteristics of marble damaged by pre-peak unloading. IOP Conf. Ser. Earth Environ. Sci..

[36] Fathi A., Moradian Z., Rivard P., Gérard B. (2016). Shear mechanism of rock joints under pre-peak cyclic loading condition. Int. J. Rock Mech. Min. Sci..

[37] Zuo J., Chen Y., Song H., Wei X. (2017). Evolution of Axial Cracks and Nonlinear Models Before the Peak of Coal-Rock Composite and Applications. Chin. J. Geol. Eng..

[38] Zuo J., Chen Y., Liu X. (2019). Crack evolution behavior of rocks under confining pressures and its propagation model before peak stress. J. Cent. South Univ..

[39] Xiao F., Jiang D., Wu F., Jie C. (2021). Deformation and Failure Characteristics of Sandstone Subjected to True-Triaxial Unloading: An Experimental and Numerical Study. Fatigue Fract. Eng. Mater. Struct..

[40] Cao X., Zhang H., Yu J., Qing Y. (2021). Experiment on strength and failure behavior of sandstone containing pre-existing cracks under brazilian compression. Front. Phys..

[41] Kewalramani M., Khartabil A. (2021). Porosity Evaluation of Concrete Containing Supplementary Cementitious Materials for Durability Assessment through Volume of Permeable Voids and Water Immersion Conditions. Buildings.

[42] Sun D.D., Cao Z.J., Huang C.F., Wu K., Schutter G.D., Zhang L.H. (2022). Degradation of concrete in marine environment under coupled chloride and sulfate attack: A numerical and experimental study. Case Stud. Constr. Mater..

[43] Golewski G.L. (2023). The Effect of the Addition of Coal Fly Ash (CFA) on the Control of Water Movement within the Structure of the Concrete. Materials.

[44] Ministry of Housing and Urban Rural Development of the People’s Republic of China (2013). Standard for Test Methods of Engineering Rock Mass.

[45] Itasca F.L.A.C. (2011). FLAC-Fast Lagrangian Analysis of Continua, version 7.0.

[46] Yuan T., Chen Z., Liu W.J., Zhou X.H., Tu W.P., Wang F. (2014). Application and Mechanical Model of Capillary Mechanics in the Separation Process of Superhydrophilic Membranes. J. South China Univ. Technol..

[47] Basu A., Mishra D.A., Roychowdhu K. (2013). Rock failure modes under uniaxial compression, Brazilian, and point load tests. Bull. Eng. Geol. Environ..

[48] Fatahi H., Hossain M.M., Fallahzadeh S.H., Mostofi M. (2016). Numerical simulation for the determination of hydraulic fracture initiation and breakdown pressure using distinct element method. J. Nat. Gas Engine.

[49] Zhou Z.L., Cai X., Zhao Y., Chen L., Xiong C., Li X.B. (2016). Strength characteristics of dry and saturated rock at different strain rates. Trans. Nonferrous Met. Soc. China.

[50] Zhuang L., Kim K., Diaz M., Yeom S. (2020). Evaluation of water saturation effect on mechanical properties and hydraulic fracturing behavior of granite. Int. J. Rock Mech. Min. Sci..

